# Global phosphoproteome analysis of human bone marrow reveals predictive phosphorylation markers for the treatment of acute myeloid leukemia with quizartinib

**DOI:** 10.1038/leu.2013.347

**Published:** 2014-01-10

**Authors:** C Schaab, F S Oppermann, M Klammer, H Pfeifer, A Tebbe, T Oellerich, J Krauter, M Levis, A E Perl, H Daub, B Steffen, K Godl, H Serve

**Affiliations:** 1 Evotec (München) GmbH, Am Klopferspitz 19a, Martinsried, Germany; 2 Max Planck Institute of Biochemistry, Am Klopferspitz 18, Martinsried, Germany; 3 Department of Medicine, Hematology/Oncology, Goethe University, Theodor-Stern-Kai 7, Frankfurt, Germany; 4 German Cancer Consortium (DKTK), Heidelberg, Germany; 5 German Cancer Research Center (DKFZ), Heidelberg, Germany; 6 Department of Medicine, Hematology/Oncology, Medizinische Hochschule Hannover, Hannover, Germany; 7 Sidney Kimmel Comprehensive Cancer Center, Johns Hopkins University, Baltimore, MD, USA; 8 Hematologic Malignancies Program, Abramson Cancer Center, University of Pennsylvania, Philadelphia, PA, USA

Treatment with inhibitors of the receptor tyrosine kinase FLT3 are currently studied as promising therapies in acute myeloid leukemia (AML). However, only a subset of patients benefit from these treatments and the presence of activating mutations within FLT3 can predict response to a certain extent only. AC220 (quizartinib) is an example of a potent FLT3 inhibitor^1^ that was studied in a recent phase II open-label study in patients with relapsed/refractory AML. The complete remission rate (including CRp and CRi) in FLT3-ITD-positive patients was 54% (50/92) and the corresponding partial remission rate (PR) was 17% (16/92)^2^ Thus, although the FLT3-ITD mutation status correlates with response, the error rate in stratification of patients into responders and non-responders is high, as still 29% of the FLT3-ITD-positive patients failed to respond. Exclusion of FLT3-ITD-negative patients from AC220 treatment also seems critical, as the total response rate (CR+PR) in FLT3-ITD-negative patients is substantially lower (41%, 17/41). As AC220 is a tyrosine kinase inhibitor, we hypothesized that investigating phosphorylation-based signaling on a system-wide scale in AML cells allows for identification of markers enabling more accurate prediction of therapy response as compared to commonly used genetic markers. Hence, we applied quantitative mass spectrometry to decipher a multivariate phosphorylation site marker, which we refer to as phospho-signature, in patient-derived AML blasts that might be useful as predictive biomarkers for AC220 treatment.

We first collected bone marrow aspirates of 21 patients enrolled in the phase II clinical trial of AC220 monotherapy in AML (ACE, NCT00989261) with FLT3-ITD before treatment (Supplementary Table 1). We processed the aspirates according to a previously established sample preparation workflow (Figure 1 and Supplementary Methods). Twelve of the twenty-one samples were processed at the beginning of this study (training group) and were used to generate a training data-set for phospho-signature identification. Nine additional samples were processed toward the end of this study and were used for validating the phospho-signature (validation group). All patients with CR or PR were counted as responder in our study (6/12 in the training subgroup and 6/9 in the validation subgroup).

To monitor quantitatively the phospho-proteomes of the patient-derived AML blasts, we used super-SILAC in combination with quantitative mass spectrometry (see Figure 1 and Supplementary Methods). Data analysis was finally performed by using the MaxQuant software^3^ and further bioinformatics tools as outlined below. In total, 13 236 phospho-sites were identified in the training group. Of these, 7831 were confidently assigned to specific serine, threonine or tyrosine residues (class I sites).

We first investigated whether we can identify differentially regulated phospho-sites when comparing responder and non-responder samples (Figure 2a). Only class I sites quantified in at least two thirds of the experiments were used (2119 sites with approximately 10.6% missing values on average). Indeed, application of the mean-rank test^4^ revealed three significantly different sites at a false-discovery rate of 10% (see Supplementary Table 2). The first regulated site (S160) is located on the endonuclease/exonuclease/phosphatase family domain-containing protein 1 (EEPD1). The protein carrying the second phosphorylation site (S630) was B-cell lymphoma/leukemia 11A (BCL11A), which functions as a myeloid and B-cell proto-oncogene and may play a role in leukemogenesis and hematopoiesis.^5^ Furthermore, the expression of BCL11A is associated with a poor outcome of AML patients.^6^ The third phosphorylation site (S333) is located on Ran-binding protein 3 (RANBP3). RANBP3 mediates nuclear export of Smad2/3 and thereby inhibits TGF-β signaling.^7^ Furthermore, the Ras/ERK/RSK and the PI3K/AKT signaling pathways regulate the activity of RANBP3.^8^ Both the pathways are activated in FLT3-ITD-positive cells.^9^ To our knowledge, no function has been described for these phospho-sites in AML so far. Interestingly, other phosphorylation events that are downstream of FLT3-ITD, such as phosphorylation of Y694 in STAT5A, were not differentially regulated between the responder and the non-responder group (Supplementary Figure 1). Hence, it appears that only certain signaling pathways downstream of FLT3-ITD are differentially regulated between responders and non-responders and these pathways might contribute to resistance-mediating bypass signaling.

Next, we sought to identify a phospho-signature that allows prediction of responsiveness using a supervised machine learning approach. We therefore applied our previously described workflow for detecting phospho-signatures.^10^ A detailed description of the bioinformatics workflow is outlined in the Supplementary Methods.

The resulting final phospho-signature consisting of five phosphorylation sites strongly separates the classes of responder and non-responder samples (Figures 2b and c, Supplementary Figure 2 and Supplementary Table 2). Three of the five phosphorylation sites (EEPD1-S160, BCL11A-S630, RANBP3-S333) were already identified as significantly regulated between responder and non-responder samples. The fourth phosphorylation site (S961) is located on the x-linked retinitis pigmentosa GTPase regulator (RP3). RP3 is predicted to be a guanine-nucleotide releasing factor and has a role in ciliogenesis.^11^ Lamins A/C (LMN1), which harbored the fifth site (S458) from the phospho-signature, form the nuclear lamina and has an important role in cell cycle-dependent regulation of nuclear structure and gene transcription.^12^ All five sites were identified and localized with high confidence (*P*>0.98, see MS^2^ spectra in Supplementary Figure 3).

The prediction performance of the phospho-signature was determined by leave-one-out cross-validation. In each iteration of cross-validation, the selection of phospho-site features and the training of a support vector machine is repeated on the training set reduced by the respective test sample. Notably, all samples except one sample (AML008) were correctly classified (Figure 2b), corresponding to a prediction accuracy of 92%. Similarly the area under the receiver operating characteristic curve is 88%. Although in case of AML008 no remission was observed, this patient also harbored a FLT3-TKD (D835) mutation at disease progression following 4 months of therapy, indicating FLT3 was inhibited as the mechanism for clinical response, albeit less than protocol defined PR.

We finally applied the identified phospho-signature to test its predictive power on nine additional validation samples (Figure 2d). These samples were processed independently of the training samples. Notably, seven out of the nine samples were predicted correctly, just one responder (AML031) and one non-responder (AML033) were misclassified. AML033 was a borderline candidate. Notably, the patient had FLT3-ITD-positive cells that were sensitive and cleared by the drug treatment. However, the patient eventually progressed with a FLT3 wild-type clone. Even if taking this ambiguous call into account, the resulting sensitivity on the validation samples is 83% and the specificity is 67%. The corresponding accuracy is 78% and therefore comparable to the accuracy determined in cross-validation. This is a promising result as the validation subgroup differed from the training subgroup both in terms of the source and in terms of the day of processing.

Differences in phosphorylation of a specific site may be caused by either a difference in phosphorylation site stoichiometry, a difference in expression of the corresponding protein, or by a combination of both. In order to distinguish between these three possibilities, we analyzed the proteome of six validation samples (Supplementary Figure 4). For two of the five signature proteins (EEPD1 and LMN1), we could quantify the predictive phosphorylation site and protein abundance in at least 2/3 of the samples. LMN1 shows a very high correlation between phosphorylation and protein expression (Pearson correlation *r*=0.92, *P*=0.03). The correlation for EEPD1 is smaller and not significantly different from 0 (*r*=0.70, *P*=0.18) due to one outlier sample (Supplementary Figure 4A). Furthermore, although we enriched for phosphorylated peptides, we identified and quantified non-phosphorylated peptides of LMN1 in almost all training and validation samples. We could therefore correlate the phosphorylation of LMN1 with its expression in these samples (Supplementary Figure 4B) and again obtained a high correlation (*r*=0.86, *P*=2.5 × 10^−6^).

These results show the utility of a global and unbiased analysis to enable the identification of non-obvious but highly predictive markers that have no known association with the drug's main target. For clinical application of the biomarker signature, it would be sufficient to detect and quantify five phosphorylation sites. Notably, economic targeted detection methods, such as immunological methods or the mass spectrometry-based multiple reaction monitoring^13^ could be applied instead of global analysis strategies. Such targeted methods can reproducibly detect and quantify given peptides from relatively low sample amounts and can be routinely applied to large number of samples. We also showed that at least one of the phosphorylation markers, LMN1 (S458), strongly correlates with the expression of the corresponding protein. This creates the further option to measure LMN1 protein expression rather than performing targeted phosphorylation site analysis.

In summary, phosphoproteomic analyses of primary AML bone marrow by high-resolution quantitative mass spectrometry is feasible and offers the opportunity to discover posttranslational modifications as pre-therapeutic response parameters. A signature consisting of five phosphorylation sites predicted the response to treatment of AML patients with AC220.

## Figures and Tables

**Figure 1:**
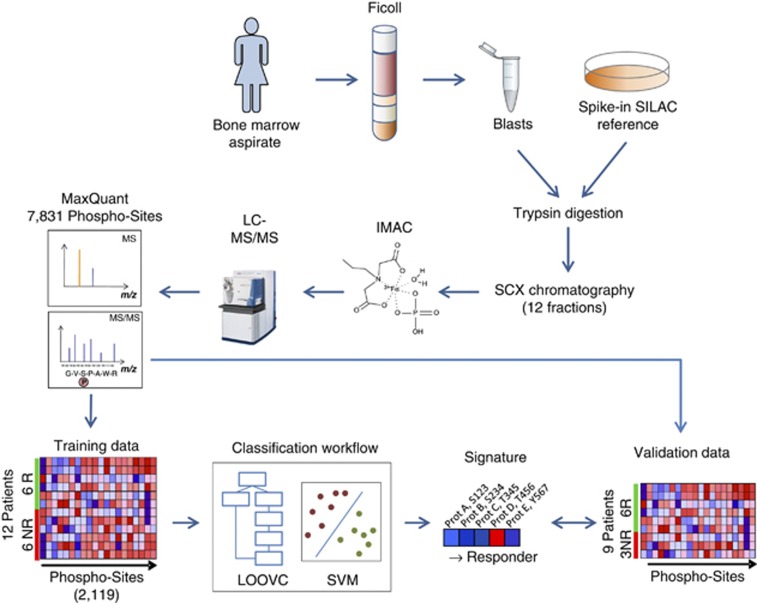
Workflow of processing bone marrow aspirates and global quantitative phosphoproteome analysis. The leukemia cells were isolated using density-gradient centrifugation and stored as vital cells for further processing at −80 °C. Equal amounts of lysates from blasts and Super-SILAC-standard were mixed. Proteins were extracted and digested with trypsin. The resulting peptides were separated into 12 fractions by strong cation exchange (SCX) chromatography and the phosphopeptides were enriched using immobilized metal affinity chromatography (IMAC). High-resolution LC-MS/MS data were processed using the MaxQuant software. Data from 12 patients (six responders and six non-responders) were used in the classification workflow for selection of five predictive phosphorylation sites (phospho-signature) and for training of a support vector machine. Classification accuracy was estimated with leave-one-out cross-validation. Finally, the signature was applied to nine independent validation samples.

**Figure 2:**
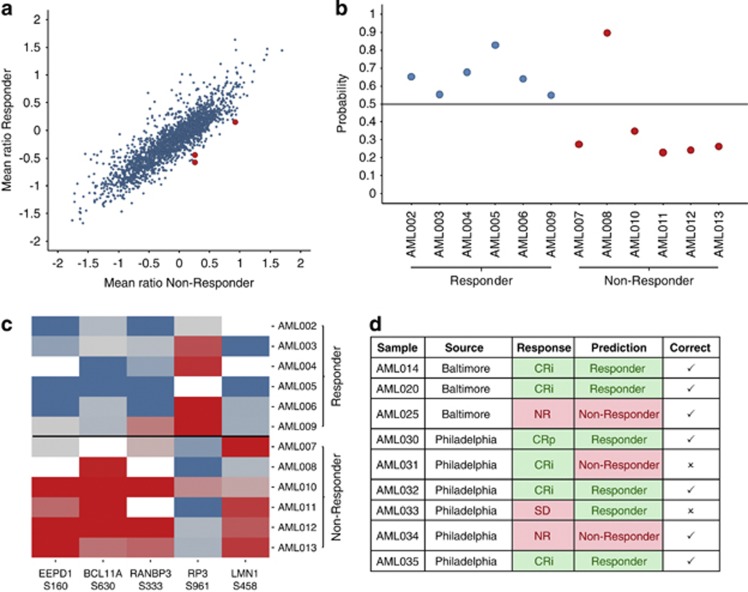
Identification of predictive phospho-signature. (**a**) Scatter plot showing the mean log-ratios (AML sample vs spike-in SILAC reference) for the responder (*y* axis) and non-responder (*x* axis) samples. Each dot represents one phosphorylation site. The three significantly differential sites are marked red. (**b**) Cross-validation results represented by the probability for assignment to the responder class. Responders (left half) are predicted correctly if they are assigned a probability >0.5; non-responder (right half) are correct if they are assigned a probability <0.5. (**c**) Heat map of the final five selected phosphorylation sites. Rows are the 12 training sample, columns are the phospho-sites ordered by their importance ranks (left is the best). Red indicates up-, blue downregulation, gray no regulation. Missing values are colored white. (**d**) Validation results of nine independent patients. The true and predicted responses are compared.

## Notes

CS, AT, HD and KG are employees and stock owners of Evotec. FSO and MK are employees of Evotec. AEP is consultant of Ambit Biosciences (unpaid) and Astellas (paid). ML is consultant of Astellas. The remaining authors declare no competing financial interest.
