# Supplementary material for: Global phosphoproteome analysis of human bone marrow reveals predictive phosphorylation markers for the treatment of acute myeloid leukemia with quizartinib
Source: Leukemia. 2014 Jan 10;28(3):716–9. doi: 10.1038/leu.2013.347 (PMC3948157; doi:10.1038/leu.2013.347)
Supplement: Supplementary Information 1 [file leu2013347x1.pdf]

Online Supplements

Global Phosphoproteome Analysis of Human Blasts Reveals Predictive Phosphorylation Markers for the Treatment of Acute Myeloid Leukemia with Quizartinib

Christoph Schaab, Felix S. Oppermann, Martin Klammer, Heike Pfeifer, Andreas Tebbe, Thomas Oellerich, Jürgen Krauter, Mark Levis, Alexander E. Perl, Henrik Daub, Björn Steffen, Klaus Godl, Hubert Serve

Supplemental Methods

Specimen collection and processing

Bone marrow aspirates of 21 patients suffering from AML were collected. 21 patients were enrolled in the phase II clinical trial of AC220 monotherapy in AML with FLT3-ITD mutations at the Goethe University (Frankfurt, Germany), the Medizinische Hochschule (Hannover, Germany), the Johns Hopkins University (Baltimore, Maryland), and the University of Pennsylvania (Philadelphia, Pennsylvania). Details on the clinical trial (ACE, NCT00989261) are reported elsewhere (ref. 1, 2). Samples were collected pre-treatment. All patients gave informed consent according to the Declaration of Helsinki to participate both in the clinical trials and the collection of samples. Use of bone marrow aspirates was approved by the respective local ethical committee at each individual institution.

The patients were divided into two subgroups. The first subgroup of in total 13 patients consists of samples from Goethe University, from Medizinische Hochschule, and a first set of 5 samples from University of Pennsylvania. These samples were used for training. The subgroup also contains the patient who was not enrolled and for whom the AC220-response is thus unknown. The second subgroup of in total 9 pa-

tients consists of samples from Johns Hopkins University and a second set of 6 samples from University of Pennsylvania. These samples were used for validation. Both subgroups were processed in separate batches. All clinics followed a standard operating procedure for preparation of the bone marrow aspirates. In brief, a maximum of 8 ml bone marrow aspirate was collected in 2 ml of ethylenediaminetetraacetic acid (EDTA) or 10% heparin, processed by FICOLL separation and then stored in 10%DMSO/10% FCS in liquid nitrogen.

The clinical protocol defined responses using International Working Group responses (ref. 3), with modification that CRi and PR responses were defined as the absence of circulating or extramedullary blasts and the presence of <5% marrow blasts or 5-25% marrow blasts, respectively. Additionally, transfusion independence was not required for either CRi or PR responses. For the purpose of our phosphoproteomic analysis, patients with complete remission (CR), complete remission with incomplete haematological recovery (CRi), complete remission with incomplete platelate recovery (CRp), and partial remission (PR) were counted as responder. Patients with stable disease (SD) or no response were counted as non-responder.

Spike-in reference

The human AML cell lines OCI-M1, NB4, and MV4-11 were chosen as Super-SILAC reference (ref. 4). OCI-M1 and NB-4 were obtained from Christian Junghanß' group (University Rostock, Germany). MV4-11 was obtained from the DSMZ – Deutsche Sammlung von Mikroorganismen und Zellkulturen (Braunschweig, Germany). NB-4 and MV4-11 were cultivated in RPMI, 10% foetal bovine serum, 2 mM glutamine, 1 mM sodium pyruvate and penicillin/streptomycin (PAA, Cölbe, Germany). OCI-M1 was cultivated in IMDM, 10% foetal bovine serum, 2 mM L-glutamine, and penicillin/streptomycin (PAA, Cölbe, Germany). Metabolic labelling of the cell lines was performed using SILAC (stable isotope labelling by amino acids in cell culture (ref. 5)). Cells were cultivated in media containing SILAC-RPMI or IMDM (PAA) and dialysed FBS (PAA). L-lysine and L-arginine were replaced by heavy isotope-labelled L-$^{13}C_6{}^{15}N_2$-lysine (Lys-8) and L-

$^{13}C_6{}^{15}N_4$-arginine (Arg-10). Isotope-labelled amino acids were purchased from Cambridge Isotope Laboratories (Andover, MA, USA). Cells were cultivated for a minimum of six cell-doubling to obtain incorporation efficiencies for the labelled amino acids of at least 95%. The labelled cells were lysed, aliquoted, and stored at -80°C.

Phosphoproteomics workflow

Viable stocks of frozen AML cells were thawed on ice, centrifuged (3 min, 2,000 rpm, 4°C) and then lysed in ice-cold lysis buffer (8 M urea, 50 mM Tris pH 8.2, 10 mM sodium pyrophosphate, 5 mM EDTA, 5 mM EGTA, 10 mM sodium fluoride, 10 mM β-glycerophosphat, 2 mM sodium orthovanadate, phosphatase inhibitor cocktail 2 and 3 (Sigma, 1:100 (v/v)) and Complete Protease Inhibitor Cocktail Tablets (Roche). After sonication the cell debris was removed by centrifugation (10 min at 13,000 rpm, 4°C) and the protein concentration was determined utilizing the Bradford Protein Assay (BIO-RAD). Equal protein amounts of the Super-SILAC reference were added and subsequently subjected to reduction (10 mM dithiothreitol, 30 min 37°C) and alkylation (50 mM chloroacetic acid, 30 min RT). The alkylation reaction was quenched by adding 20 mM DTT. Proteins were initially digested with lysyl enpopeptidase (Wako, 1:200 (w/w)) for 4 hours then diluted 5-times with 20 mM Tris pH 8.2 prior to overnight proteolytic cleavage with trypsin (Promega, 1:100 (w/w)). The peptide mixtures were acidified by addition of TFA to a final concentration of 0.5 % and subsequently desalted via C18 Sep-Pak columns (Waters). Peptides were eluted with 50% acetonitrile, 0.5% acetic acid, snap frozen in liquid nitrogen and lyophilized.

Phosphorylated and non-phosphorylated peptides were initially fractionated by strong-cation-exchange (SCX) chromatography based on a previously described protocol (ref. 6) using a PolySULFOETHYL A column (200x2.1 mm, 200 Å pore size and 5 mm particle size; PolyLC) operated with an Äkta Purifier system (GE Healthcare). Briefly, the dried peptides were reconstituted in 100 µl SCX buffer A (5 mM K_2HPO_4, pH 2.7, 30% acetonitrile) and loaded onto the SCX column. The peptides were separated by a linear gradient from 0 to 25% SCX buffer B (buffer A supplemented with 500 mM KCl) over 32 min at a flow rate of 0.5

ml/min. Fractions of 1 ml were collected across the gradient and combined to 12 distinct samples. These samples were then lyophilized and the dried peptides were subsequently reconstituted in 1 ml of 0.1% TFA and desalted using C18 reversed phase cartridges (Waters) as described by the manufacture. The desalted peptides were eluted with 50% acetonitrile, 0.5% acetic acid and lyophilized again.

Dried phosphopeptides of each fraction were reconstituted in IMAC binding buffer (40% acetonitrile, 25 mM formic acid) and phosphopeptides were captured using PHOS-Select® iron affinity beads (Sigma) based on the protocol by Villen *et.al.* (ref. 6). Briefly, 5 µl of equilibrated IMAC beads were loaded onto in-house made IMAC-C18-STAGE-Tips (IMAC-StageTips) and the peptide samples were loaded by centrifugation (3,000 rpm). After washing with 1 % formic acid, phosphopeptides were eluted onto the C18 frit with 500 mM K_2HPO_4. Phosphopeptides were then eluted with 50% acetonitrile, 0.5% acetic acid after additional washing steps with 0.1 % TFA and 0.5 % acetic acid and dried in a vacuum concentrator (Eppendorf). For MS-anaylsis phosphopeptides were reconstituted in 0.5% acetic acid.

LC-MS/MS Analysis

All LC-MS/MS analyses were performed on an LTQ-Orbitrap Velos (Thermo Fisher Scientific). The samples were loaded by an Proxeon easy nano LC II system (Thermo Fisher Scientific) on a 15 cm fused silica emitter (New Objective) packed in-house with reversed phase material (Reprusil-Pur C18-AQ, 3 µm, Dr. Maisch GmbH) at a maximum pressure of 275 bar. The bound peptides were eluted by a gradient from 10% to 60% of solvent B (80% acetonitrile, 0.5% acetic acid) at a flow rate of 200 nl/min and sprayed directly into the mass spectrometer by applying a spray voltage of 2.2 kV using a nanoelectrospray ion source (ProxeonBiosystems). The mass spectrometer was operated in the data dependent mode to automatically switch between MS and MS/MS acquisition. To improve mass accuracy in the MS mode, the lock-mass option was enabled as described (ref. 7). Full scans were acquired in the orbitrap at a resolution R = 60,000 and a target value of 1,000,000 ions. The fifteen most intense ions detected in the MS scan were selected for collision induced dissociation in the LTQ at a target value of 5000 ion counts. The

resulting fragmentation spectra were also recorded in the linear ion trap. To improve complete dissociation of phosphopeptides, the multi-stage activation option was enabled for all MS-analyses of phosphopeptide-enriched samples by applying additional dissociation energy on potential neutral loss fragments (precursor ion minus 98, 49 and 32.7 m/z) (ref. 8). Ions that were once selected for data dependent acquisition were dynamically excluded for 90 sec for further fragmentation. General used mass spectrometric settings were: spray voltage, 2.2 kV; no sheath and auxiliary gas flow; heated capillary temperature, 230°C; normalized collision energy, 35% and an activation q = 0.25.

MaxQuant analysis

MS raw files from the training and the validation subgroup were processed separately with MaxQuant (version 1.2.2.2) (ref. 9) applying the Andromeda search engine (ref. 10). The human UNIPROT database (version: 08.2011) was used comprising 125,676 database entries including the UNIPROT splice variants database. The minimal peptide length was set to 6 amino acids, trypsin was selected as proteolytic enzyme and maximally 2 missed cleavage sites were allowed. Carbamidomethylation of cysteine residues was set as fixed modification while oxidation of methionine, protein *N*-acetylation as well as phosphorylation of serine, threonine and tyrosine residues was allowed as variable modifications. As MaxQuant automatically extracts isotopic SILAC peptide doublets, the corresponding isotopic forms of lysine and arginine were automatically selected. The maximal mass deviation of precursor and fragment masses was set to 20 ppm and 0.5 Da before internal mass recalibration by MaxQuant. A false discovery rate (FDR) of 0.01 was selected for proteins, peptides, and phosphorylation sites. The MaxQuant results were uploaded to the MaxQB database (version 2.9) (ref. 11) for further analysis.

The regulation of a phosphosite is provided as ratio of the site's abundance between the spike-in SILAC reference (heavy) and the bone marrow samples (light). The normalized ratios provided by MaxQuant were log-transformed (base 10) for further analysis. Sites that satisfy the constraints Localization Probability>= 0.75 and Score Diff>=5 were considered to be sufficiently reliable (class I sites (ref. 12)). Fur-

thermore, sites that are flagged as Reverse or Contaminant hits were excluded. The identification and quantification data for all class I sites are accessible in Suppl. Information 1 and 2.

Identification and validation of phospho-signature

The statistical analysis of the data was performed in Matlab (The Mathworks, Natick, MA). The Mean-Rank test (ref. 13) was applied to find differentially abundant phosphorylation sites between two groups of samples. The Mean-Rank test is more powerful than tests based on the parametric or non-parametric t-test or Wilcoxon rank-sum test, when only few replicates are available. It controls for the FDR without requiring additional correction for multiple hypothesis testing. For this analysis only phosphosites with values in at least two thirds of the experiments in each group were considered. The FDR was set to 0.10.

The details of the workflow for identification of a predictive phospho-signature were described elsewhere (ref. 14). In brief, the Mean-Rank test was used in combination with the ensemble feature selection method (ref. 15). The number of features was fixed to five. The selected features were used to train a SVM with linear kernel and the parameter C=1. Missing data in the training samples were imputed by the mean of the respective class. The prediction accuracy and the AUROC were determined on the training set by leave-one-out cross-validation. Missing data in the test sample were imputed by the mean of the two class means.

Prediction probabilities were calculated from a sigmoid model that was fitted to the SVM output of the respective training data (ref. 16). In the sigmoid function $p_i = \frac{1}{1+\exp(Af_i + B)}$, where p_i is the probability of the sample being a non-responder and f_i the SVM output, only parameter *A* was optimized. Parameter *B* was fixed to 0, so that points on the separating hyper plane are assigned a probability of 0.5.

The complete training data set was used to select five predictive features and to train the final SVM, which was then applied to the classification of new samples from the validation set. Missing data were again imputed by the mean of the two class means.

Supplemental Figures

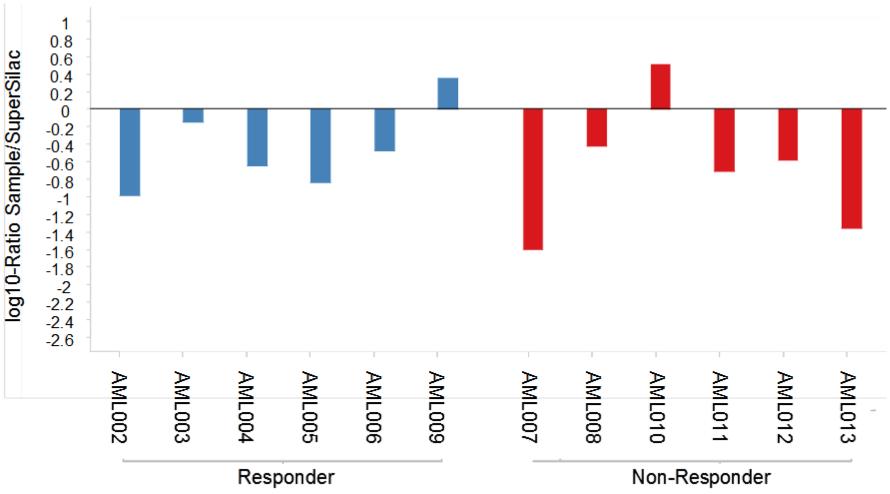

Suppl. Figure 1: Log-ratios for Y694 (STAT5A) across all training samples.

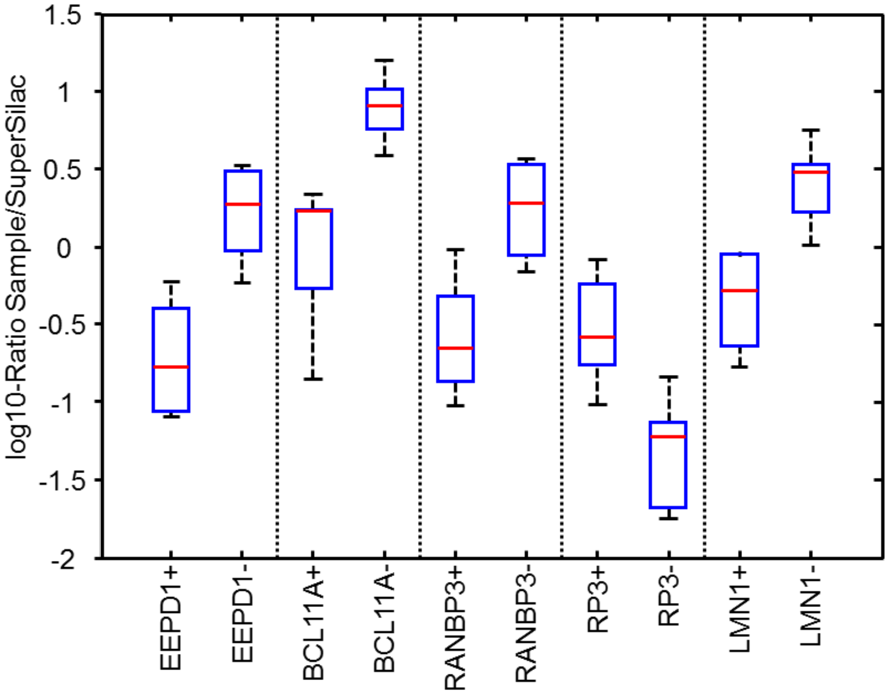

Suppl. Figure 2: Final phospho-signature consisting of 5 phosphosites. Each pair of boxes corresponds to one phosphosite. The left (right) box represents the responder (non-responder) samples. On each box, the central mark is the median, the edges of the box are the 25th and 75th percentiles, the whiskers extend to the most extreme data points not considered outliers, and outliers are marked individually with crosses.

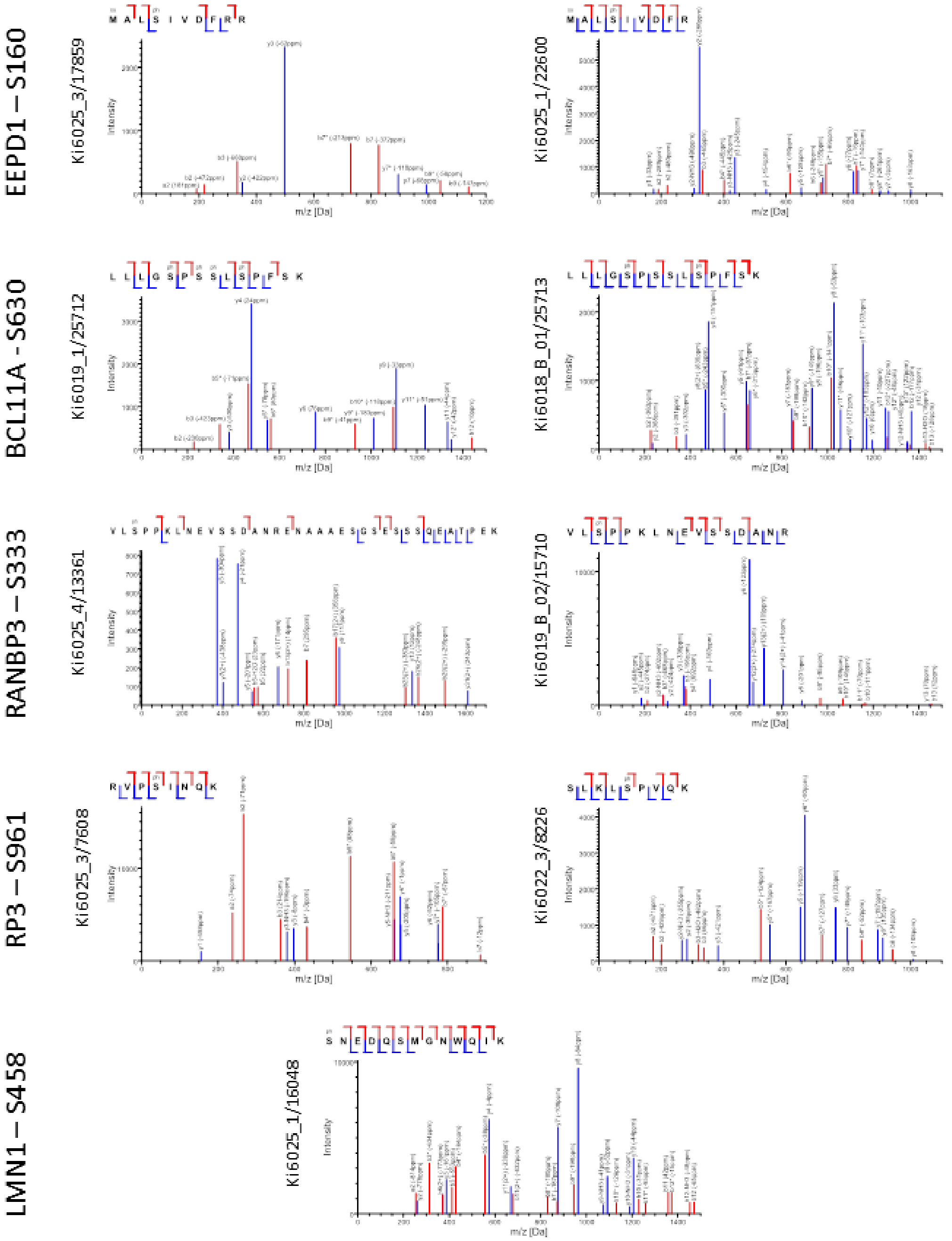

Suppl. Figure 3: MS^2 spectra for best localization (left column) and best identification (right column) evidences for the 5 signature phosphorylation sites. The name of the raw file and the MS^2 scan number is given on the left of each spectrum. Each peak is annotated with the ion type and the mass deviation from the theoretical mass in part per million (ppm). The detected b-ions (red) and y-ions (blue) are also depicted with respect to the modified peptide sequence. For LMN1 the best localization and identification evidences are identical.

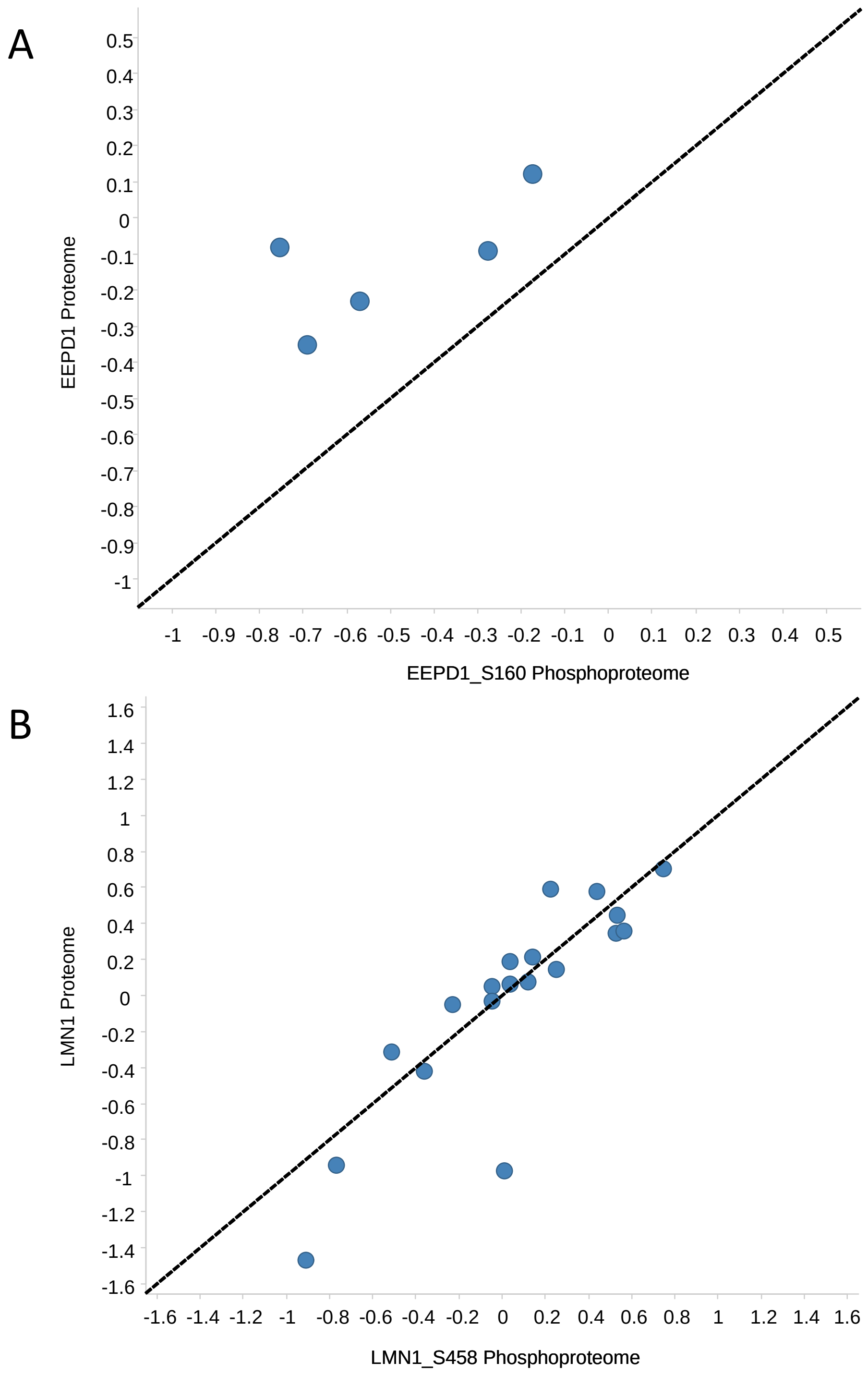

Suppl. Figure 4: Correlation between phosphorylation and protein expression. **A:** EEPD1 (S160) across six validation samples. **B:** LMN1 (S458) across all samples.

Supplemental Tables

Suppl. Table 1: Collection of analysed AML samples. CR: complete remission; CRi: CR with incomplete hematological recovery; CRp: CR with incomplete platelet recovery; PR: partial remission; SD: stable disease.

Sample ID	Source	Patient Id	Subgroup	AC220 Sensitivity	Class	Protein [µg]
AML002	Frankfurt	F32490	training	PR	+	209
AML003	Frankfurt	F32530	training	CRp	+	386
AML004	Frankfurt	F33031	training	CRi	+	123
AML005	Philadelphia	1009-018	training	CRi	+	800
AML006	Philadelphia	1009-007	training	CRi	+	230
AML007	Philadelphia	1009-002	training	SD	-	1020
AML008	Philadelphia	1009-021	training	SD	-	1720
AML009	Philadelphia	1009-014	training	CRi	+	456
AML010	Hannover	L864VR	training	SD	-	80
AML011	Hannover	M212ZM	training	SD	-	108
AML012	Hannover	L927C	training	SD	-	72
AML013	Hannover	M83BB	training	SD	-	270
AML014	Baltimore	1005-017	validation	CRi	+	400
AML020	Baltimore	1005-018	validation	CRi	+	400
AML025	Baltimore	1005-019	validation	SD	-	350
AML030	Philadelphia	1009-003	validation	CRi	+	169
AML031	Philadelphia	1009-009	validation	CRi	+	459
AML032	Philadelphia	1009-011	validation	CRi	+	261
AML033	Philadelphia	1009-016	validation	SD	-	282
AML034	Philadelphia	1009-015	validation	NR	-	377
AML035	Philadelphia	1009-019	validation	CRi	+	1254

Suppl. Table 2: Phosphorylation sites of the final phospho-signature. Median diff is the difference of the median log ratios of the responder samples and the median of the non-responder samples. SV weight is the weight of the respective feature in the support-vector-machine.

Uniprot id	Gene name	Site	Median diff (Log10)	SV weight
Q7L9B9	**EEPD1**	**S160**	**-1.05**	**-0.75**
Q9H165	**BCL11A**	**S630**	**-0.68**	**-0.54**
Q9H6Z4	**RANBP3**	**S333**	**-0.94**	**-0.31**
Q92834	**RP3**	**S961**	**0.64**	**+0.88**
P02545	**LMN1**	**S458**	**-0.76**	**-0.75**

References

1. Cortes JE, Perl AE, Dombret H, Kayser S, Steffen B, Rousselot P, et al. Final Results of a Phase 2 Open-Label, Monotherapy Efficacy and Safety Study of Quizartinib (AC220) in Patients ≥ 60 Years of Age with FLT3 ITD Positive or Negative Relapsed/Refractory Acute Myeloid Leukemia Blood (ASH Annual Meeting Abstracts). 2012;120:Abstract 48.
2. Levis MJ, Perl AE, Dombret H, Döhner H, Steffen B, Rousselot P, et al. Final Results of a Phase 2 Open-Label, Monotherapy Efficacy and Safety Study of Quizartinib (AC220) in Patients with FLT3-ITD Positive or Negative Relapsed/Refractory Acute Myeloid Leukemia After Second-Line Chemotherapy or Hematopoietic Stem Cell Transplantation. Blood (ASH Annual Meeting Abstracts). 2012;120:673.
3. Cheson BD, Bennett JM, Kopecky KJ, Buchner T, Willman CL, Estey EH, et al. Revised recommendations of the International Working Group for Diagnosis, Standardization of Response Criteria, Treatment Outcomes, and Reporting Standards for Therapeutic Trials in Acute Myeloid Leukemia. Journal of clinical oncology : official journal of the American Society of Clinical Oncology. 2003;21(24):4642-9.
4. Geiger T, Cox J, Ostasiewicz P, Wisniewski JR, Mann M. Super-SILAC mix for quantitative proteomics of human tumor tissue. Nature methods. 2010;7(5):383-5.
5. Ong SE, Blagoev B, Kratchmarova I, Kristensen DB, Steen H, Pandey A, et al. Stable isotope labeling by amino acids in cell culture, SILAC, as a simple and accurate approach to expression proteomics. Mol Cell Proteomics. 2002;1(5):376-86.
6. Villen J, Gygi SP. The SCX/IMAC enrichment approach for global phosphorylation analysis by mass spectrometry. Nat Protoc. 2008;3(10):1630-8.
7. Olsen JV, de Godoy LM, Li G, Macek B, Mortensen P, Pesch R, et al. Parts per million mass accuracy on an Orbitrap mass spectrometer via lock mass injection into a C-trap. Mol Cell Proteomics. 2005;4(12):2010-21.
8. Schroeder MJ, Shabanowitz J, Schwartz JC, Hunt DF, Coon JJ. A neutral loss activation method for improved phosphopeptide sequence analysis by quadrupole ion trap mass spectrometry. Anal Chem. 2004;76(13):3590-8.
9. Cox J, Mann M. MaxQuant enables high peptide identification rates, individualized p.p.b.-range mass accuracies and proteome-wide protein quantification. Nat Biotechnol. 2008;26(12):1367-72.
10. Cox J, Neuhauser N, Michalski A, Scheltema RA, Olsen JV, Mann M. Andromeda: a peptide search engine integrated into the MaxQuant environment. Journal of proteome research. 2011;10(4):1794-805.
11. Schaab C, Geiger T, Stoehr G, Cox J, Mann M. Analysis of high accuracy, quantitative proteomics data in the MaxQB database. Mol Cell Proteomics. 2012;11(3):M111 014068.
12. Olsen JV, Blagoev B, Gnad F, Macek B, Kumar C, Mortensen P, et al. Global, in vivo, and site-specific phosphorylation dynamics in signaling networks. Cell. 2006;127(3):635-48.
13. Klammer M, Dybowksi JN, Hoffmann D, Schaab C. Identification of Significant Features by a Global Mean Rank Test. submitted to BMC Bioinformatics2013.
14. Klammer M, Kaminski M, Zedler A, Oppermann F, Blencke S, Marx S, et al. Phosphosignature predicts dasatinib response in non-small cell lung cancer. Molecular & cellular proteomics : MCP. 2012;11(9):651-68.
15. Abeel T, Helleputte T, Van de Peer Y, Dupont P, Saeys Y. Robust biomarker identification for cancer diagnosis with ensemble feature selection methods. Bioinformatics. 2010;26(3):392-8.
16. Lin H-T, Lin C-J, Weng RC. A note on Platt's probabilistic outputs for support vector machines. Machine Learning. 2007;68(3):267-76.
